# Nutritional status according to the mini nutritional assessment (MNA)® as potential prognostic factor for health and treatment outcomes in patients with cancer – a systematic review

**DOI:** 10.1186/s12885-020-07052-4

**Published:** 2020-06-26

**Authors:** G. Torbahn, T. Strauss, C. C. Sieber, E. Kiesswetter, D. Volkert

**Affiliations:** 1 Institute for Biomedicine of Aging, Friedrich-Alexander-Universität Erlangen-Nürnberg, Kobergerstr. 60, 90408 Nuremberg, Germany; 2 Kantonsspital Winterthur, Brauerstrasse 15, 8400 Winterthur, Switzerland

**Keywords:** Neoplasms, Nutritional status, Malnutrition, Nutrition assessment, Prognosis, Systematic review

## Abstract

**Background:**

Patients with cancer have an increased risk of malnutrition which is associated with poor outcome. The Mini Nutritional Assessment (MNA®) is often used in older patients with cancer but its relation to outcome is not known.

**Methods:**

Four databases were systematically searched for studies relating MNA-results with any reported outcome. Two reviewers screened titles/abstracts and full-texts, extracted data and rated the risk of bias (RoB) independently.

**Results:**

We included 56 studies which varied widely in patient and study characteristics. In multivariable analyses, (risk of) malnutrition assessed by MNA significantly predicts a higher chance for mortality/poor overall survival (22/27 studies), shorter progression-free survival/time to progression (3/5 studies), treatment maintenance (5/8 studies) and (health-related) quality of life (2/2 studies), but not treatment toxicity/complications (1/7 studies) or functional status/decline in (1/3 studies). For other outcomes – length of hospital stay (2 studies), falls, fatigue and unplanned (hospital) admissions (1 study each) – no adjusted results were reported. RoB was rated as moderate to high.

**Conclusions:**

MNA®-result predicts mortality/survival, cancer progression, treatment maintenance and (health-related) quality of life and did not predict adverse treatment outcomes and functional status/ decline in patients with cancer. For other outcomes results are less clear. The moderate to high RoB calls for studies with better control of potential confounders.

## Background

Cancer is the second leading cause of death of non-communicable diseases worldwide [1]. Its prevalence increased by 25.4% between 2007 and 2017, and population ageing contributed about 22% to this increase [1]. Prevalence and incidence of cancer in people aged 70 years and older were estimated to be about 27.1 and 9.6 million cases in 2017 [2].

Due to the effects of both, the disease and its usually intensive treatment, patients with cancer have an increased risk of malnutrition. Various cancer-related mechanisms, such as systemic inflammation [3] and hypoxic stress [4] affect the patients’ nutritional status. Patients might already present lower dietary intake before anticancer treatment [5] and in addition, side effects of anticancer therapy, e. g. loss of appetite, dry mouth or nausea that are associated with a lower energy intake [6]. The prevalence of malnutrition in patients with cancer is described by 26–42% [7–9], and varies between different operationalisations [10–12]. To better reflect the health status of an older patient before treatment decisions are made by oncologists, a (comprehensive) geriatric assessment is recommended [13–15], consisting of several domains such as functional status, cognition, comorbidity or polypharmacy and it is also recommended that it should contain a domain regarding the patients’ nutritional status assessed by validated tools such as the Mini Nutritional Assessment (MNA)® [15]. A recent study by Kenis et al. could show that components of comprehensive geriatric assessment are prognostic factors (especially functional status and nutritional status) for overall survival in patients with cancer which additionally highlights the need for nutritional assessment [16]. It was also shown, that (severe) malnutrition is independently associated with mortality risk and decreased tolerance of chemotherapy [17]. Therefore, early detection and treatment of malnutrition is recommended for the prevention of cancer-related adverse outcomes [18–20].

However, no gold standard for screening and assessment of malnutrition in cancer patients exists. Among 37 malnutrition screening and assessment methods utilized for patients with cancer in clinical practice, in a recent systematic review, the MNA scored highest for the calculated content validity [21]. This tool is validated to identify persons aged 65 years or older who are at risk of malnutrition or malnourished [22–25].

The MNA is widely used in patients with cancer of all ages [26], even though it is neither developed specifically for this disease nor for persons younger than 65 years. Both versions, the short-form (MNA-SF) and long-form (MNA-LF), are recommended for screening of nutritional status of older patients in all clinical settings [27]. For patients with cancer, the use of MNA-SF is recommended by medical oncology societies for older patients with cancer [28, 29] as well as by practicing oncologists [30]. A summary of results about the association between MNA and relevant patient outcomes is currently lacking. Thus, our aim was to systematically summarize the existing evidence regarding nutritional status according to the MNA as potential prognostic factor for health and treatment outcomes in cancer patients.

## Methods

This systematic review is reported according to the Preferred Reporting Items for Systematic Reviews and Meta-Analyses (PRISMA) statement [31]. A protocol describing the methodological procedure was prepared before the start and is available upon request.

### Systematic literature search

A systematic literature search using database specific search strategies was conducted in MEDLINE and EMBASE (via Ovid), the Cochrane Library and CINAHL (via EBSCOhost) in June 2017 for studies published in any language from 1994 (first published version of MNA) onwards. The search was updated twice, in September 2018 and March 2020. Search strategies have been developed by 1 reviewer (GT) and discussed by the working group members (GT, TS, EK and DV) and a librarian. The search strategies included a combination of keywords and MeSH−/ Emtree-terms (e.g. nutritional status, MNA, cancer) (Additional file, table 1). Additionally, reference lists of included studies were searched.

### Study selection

Original articles of longitudinal studies reporting a potential association between nutritional status assessed by MNA (any form) at baseline and any health or treatment outcome (e.g. mortality, survival, complications) at a later time point in patients of any age with any type of cancer and anticancer therapy were included. Studies with a cross-sectional design and those not using MNA-assessed nutritional status for predicting health and treatment outcomes were excluded as well as other publication types (e.g. conference abstracts or editorials). Currently, 2 forms of the MNA are available, which were both included. The short-form (SF) consisting of 6 items (A-F), first developed in 2001 [24] and revised in 2009 (range 0–14 points; 0–7 points: malnourished; 8–11 points: at risk of malnutrition and 12–14 points: normal nutritional status) [23], and the long-form (LF) or “full MNA” consisting of additional 12 items (G-R) [22, 25] (range 0–30 points; 0–17 points: malnourished; 17–23.5 points: at risk of malnutrition and 24–30 points: normal nutritional status).

Titles/abstracts and full texts were screened by 2 reviewers (GT, TS) independently. Conflicts were solved by discussion or by a third reviewer (EK).

### Data extraction

Two reviewers (GT, TS) independently extracted the following data using a piloted extraction form:

a) Study characteristics: first author, year of publication, country, sample size.

b) Participant characteristics: age, sex, type of cancer, cancer stage, anticancer therapy (e.g. chemotherapy).

c) Malnutrition screening tool and result: MNA form (MNA-SF or -LF), MNA result as reported by the authors (prevalence of malnutrition, risk of malnutrition and well-nourished patients and/or mean/median score.

d) Outcome characteristics: follow-up time, prevalence or incidence of any reported outcome at/during follow-up; results on prognostic effects (e.g. odds ratios (OR), hazard ratios (HR) for respective outcome (e.g. mortality)) from multivariable analyses.

### Assessment of risk of bias

Two reviewers (GT, EK) independently assessed the risk of bias (RoB) of each included study using a specified version of the QUIPS-tool [32] (Additional file, table 2). We predefined a set of core confounders (cancer stage, type of cancer, type of therapy, sex, age, performance status, co-morbidity) and dropped the first item ‘definition of the prognostic factor’ of the ‘*prognostic factor measurement*’ domain since we were interested in nutritional status according to MNA as the only prognostic factor. The item ‘*valid and reliable measurement of prognostic factor*’ was rated as having a low risk of bias when the study reported all 3 MNA-categories or the MNA-score.

The domains study participation, study attrition, prognostic factor measurement, outcome measurement, study confounding and statistical analysis and reporting were rated with either low, moderate or high RoB and are separately presented for each study. Conflicts were solved by discussion or a third reviewer (DV).

### Data synthesis

Reported outcomes were classified in 7 categories: (a) mortality/ poor overall survival, (b) progression-free survival and time to progression, (c) treatment maintenance or duration, (d) adverse treatment outcomes (toxicity, complications), (e) functional status / decline and (f) quality of life and (g) other outcomes.

Due to a high heterogeneity of patient populations and reported outcomes meta-analyses were not possible.

## Results

### Study selection

**Fig. 1:**
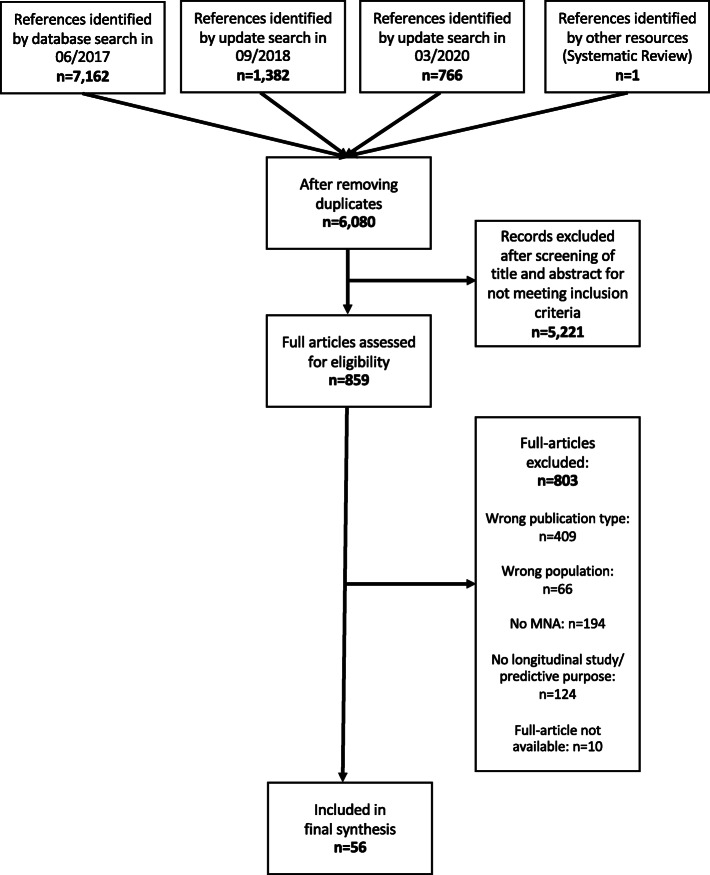
PRISMA Flow chart

After removing duplicates, we screened 6080 titles/abstracts and 859 full-texts for potential eligibility. Finally, 56 studies [16, 33–87] were included, all of them published in English language. Main reasons for exclusion were wrong publication type (e.g. conference abstract), no use of MNA, or no longitudinal study design/predictive purpose (Fig. 1).

### Study and patient characteristics

Detailed study and patient characteristics are presented in Table 1.

**Table 1:** Study and patient characteristics of included studies

Author/ Year	Country	***N***^**a**^	Age (years)^**b**^	Female (%)	Type of cancer	Cancer stage (early/ mixed/ advanced)	Anticancer therapy	MNA-form	MNA-result^**c**^			Outcome
									MN (%)	AR (%)	WN (%)	
Aaldriks 2011	NL	202	77 ± 4	55	Various	Mixed	Chemo	SF/LF^d^	2.5	29.7	64.9	Mortality^e^ Treatment maintenance
Aaldriks 2013a	NL	143	75 (70–92)	41	Colorectal	Mixed	Chemo	SF/LF^d^	28.0		72.0	Mortality Treatment maintenance
Aaldriks 2013b	NL	55	76 ± 5	96	Breast	Advanced	Chemo	SF/LF^d^	41.8		54.5	Mortality Treatment maintenance
Aaldriks 2015	NL	44	78 (70–86)	57	Various	Mixed	Chemo	SF/LF^d^	34.1		65.9	Mortality Treatment maintenance
Aaldriks 2016	NL	494	75 (70–92)	51	Various	Mixed	Chemo	SF/LF^d^	35.2		64.0	Mortality Treatment maintenance
Allaire 2017	Canada	144	69 ± 10	22	Bladder	Mixed	Surgery	SF	9.0	43.0	48.0	Complications
Aparicio 2018	France	102	81 (75–89)	45	Colorectal	Advanced	Chemo	SF	66.7		33.3	Mortality PFS
Araujo 2017	Brazil	52	53 (24–85)^f^	71	Various	Mixed	Chemo	LF	23.5 ± 4.2^g^			Fatigue
Baier 2016	Germany	195	75 (70–88)	31	Various	n.r.	Surgery	n.r.	n.r.	56.7^h^	n.r.	Functional status
Boulahssass 2018	France	1050	82 (70–100)	60	Various	Mixed	Various	LF	21.0	47.7	28.5	Mortality
D’Almeida 2020	Brazil	3061	73 ± 7	44	Various	Mixed	Various	SF	33.4	39.3	27.3	Length of hospital stay
Decoster 2016	Belgium	193	77 (70–89)	38	Colorectal	Mixed	Various	LF	56.0		44.0	Functional decline Toxicity
Decoster 2018	Belgium	252	77 (69–91)	39	Colorectal	Advanced	Various	LF	54.2		45.8	PFS Treatment duration
Decoster 2019	Belgium	2972	79 ± 6	57	Various	Mixed	Various	SF	79.1		21.9	Quality of Life
Dubruille 2015	Belgium	90	74 (65–89)	43	Hematological	Mixed	Chemo	LF	44.0		56.0	Mortality
Extermann 2012	US	n.a.^i^	76 (70–92)	50	Various	Mixed	Chemo	LF	25 (8–30)^g^			Toxicity
Frasca 2018	France	1264	78 ± 5	70	Various	Mixed	Various	LF	41.5		58.5	Mortality
Ghosn 2017	France	100	76 (4)^j^	47	Various	n.r.	Various	SF	n.r.	n.r.	n.r.	Mortality
Giannotti 2019	Italy	99	80 ± 6	38	Colorectal	Mixed	Surgery	LF	23.12 ± 3.31^g^			Mortality
Giannousi 2012	Greece	122	66 (37–81)	16	Lung	Advanced	Systemic	LF	9.0	60.7	30.3	Mortality
Gioulbasanis 2011a	Greece	173	65 ± 11	17	Lung	Advanced	Various	LF	26.0	46.2	27.8	Mortality TTP
Gioulbasanis 2011b	Greece	115	66 (32–86)	12	Lung	Advanced	Chemo	LF	25.2	51.3	23.5	Mortality
Gioulbasanis 2012	Greece	114	68 ± 5	11	Lung	Advanced	Systemic	LF	29.8	41.2	29.0	Mortality TTP
Gioulbasanis 2015	France, Greece	594	69 ± 10	27	Various	Advanced	Systemic	LF	12.8	49.5	37.7	Mortality
Goineau 2018	France	100	78 (75–89)	0	Prostate	Advanced	Radio	LF	2.0	98.0		Quality of Life
Gu 2015	China	300	56 ± 12	33	Renal cell	Advanced	n.r.	SF	4.0	17.3	78.7	Mortality Toxicity
Honecker 2018	Germany	160	78	0	Prostate	n.r.	Various	SF	61.6		38.4	Treatment maintenance
Hoppe 2013	France	299	77 (70–93)	41	Various	Advanced	Chemo	LF	63.2		36.8	Functional decline
Kaibori 2016	Japan	71	78 ± 5	27	Hepatocellular	n.r.	Surgery	SF	43.7		56.3	Complications
Kenig 2015	Poland	75	73 ± 6	44	Various	Various	Surgery	LF	48.0		52.0	Complications
Kenis 2017	Belgium	439	75 (70–95)	58	Various	Mixed	Various	SF	21.4	46.9	31.7	Mortality Functional decline
Kenis 2018	Belgium	Cohort A: 763	Cohort A: 76 (70–95)	Cohort A: 68	Various	Mixed	Various	SF	Cohort A: 62.3		Cohort A: 37.7	Mortality
		Cohort B: 402	Cohort B: 77 (70–95)	Cohort B: 67					Cohort B: 56.5		Cohort B: 43.5	
Kim 2014	Korea	98	n.a.^k^	31	Various	Mixed	Chemo	LF	20.4	47.0	30.6	Treatment maintenance
Kristjansson 2010	Norway	182	80	57	Colorectal	Mixed	Surgery	LF	9.5	45.6	45.0	Mortality Complications
Liuu 2020	France	1092	82 ± 5	47	Various	Mixed	Various	LF	14	50	36	Mortality Unplanned admission
Lycke 2019	Belgium	944	80 (70–99)	48	Various	Mixed	Various	SF	73		27	Mortality
Martucci 2016	Brazil	136	73 ± 7	48	Various	Mixed	n.r.	SF	29.4	41.2	29.4	Mortality
Mazzuca 2019	Italy	ProLYOtin: 22	ProLYOtin: 68 (34–83)	ProLYOtin: 32	Colorectal	Mixed	Chemo	LF	ProLYOtin: 18	ProLYOtin: 50	ProLYOtin: 32	Toxicity
		Placebo: 25	Placebo: 67 (49–85)	Placebo: 44					Placebo: 16	Placebo: 36	Placebo: 48	
Michaan 2020	Korea	120	76 ± 5	100	Gynaecologic	Mixed	Various	LF	20.4 ± 4.6^g^			Mortality
Molga 2020	Australia	98	77 (66–95)	37	Hematological	Mixed	Various	LF	27		73	Mortality Treatment maintenance
Naito 2016	Japan	93	77 (65–90)^f^	54	Non-Hodgkin Lymphoma	Mixed	Various	LF	40.9	59.1		Mortality
Osborne 2017	UK	178	74 (70–84)	0	Prostate	Mixed	Various	SF	0.0	6.7	82.6	Toxicity
Park 2015	Korea	70	74 (65–92)	46	Lymphomas	Mixed	Chemo	SF	35.7	37.1	27.1	Mortality Treatment maintenance
Quinten 2019	Belgium	741	81 (70–89)	43	Various	Mixed	Surgery	SF	74.4		25.6	Quality of Life
		683	77 (70–94)	47			Chemo		81.4		18.6	
Retornaz 2020	France	97	79 (75–83)^l^	51	Colorectal	Advanced	Chemo	SF	61.9		38.1	Mortality Toxicity
Samuelsson 2019	Sweden	49	81 (77–85)^l^	53	Colorectal	Mixed	Surgery	SF	38.8		61.2	Complications Length of hospital stay
Scholtz 2018	Germany	517	71 (68–74)^l^	32	Various	Mixed	Surgery	LF	2.3	23.8	73.9	Complications
Schütte 2015	Germany	51	66 ± 10	14	Hepatocellular	Mixed	n.r.	LF	0.0	17.3	62.7	Mortality
Shin 2012	Korea	64	71 (65–80)	25	Various	Mixed	Chemo	LF	21.9	59.4	18.7	Toxicity
Shiroyama 2017	Japan	30	76 (70–83)	13	Lung	Advanced	Chemo	SF	0.0^m^	66.7	33.3	Toxicity
Soubeyran 2012	France	348	77 (70–99)	41	Various	Mixed	Chemo	LF	64.9		35.1	Mortality
Stauder 2020	Austria	147	78 (67–98)	46	Hematological	Mixed	Various	LF	15	43	42	Mortality
van Deudekom 2019	NL	102	79 (72–85)^l^	30	Head & Neck	Mixed	Various	SF	39.2		61.8	Mortality
van der Vlies 2019	NL	99	77 (69–85)^l^	37	Various	Advanced	Chemo	LF	85.0		15.0	Treatment maintenance Toxicity
Vande Walle 2014	Belgium	937	76 (70–95)	64	Various	Mixed	n.r.	SF	18.7	45.0	36.6	Falls
Vlachostergios 2013	Greece	103	67 (32–84)	10	Lung	Advanced	Systemic	LF	30.1	39.8	30.1	Mortality PFS

Legend: ^a^Number of included patients; ^b^mean ± standard deviation (SD) or median (range); ^c^percentages may not add up to 100 due to rounding or missing values; ^d^stepwise process – patients were classified as WN when SF > 12 or LF 24–30; ^e^studies that were categorized to ‘mortality’ reported results on mortality or (poor) overall survival; ^f^mean (range); ^g^MNA-LF-score: X ± Y: mean ± SD or X (Y-Z) median (range: 0–30); ^h^”Based on literature and the distribution of the mean values in the current study population“ [23]; ^i^total: 518; ^j^median (SD); ^k^87.8 ≥ 70 years; ^l^interquartile-range (IQR); ^m^MN patients were excluded from study participation

*MNA* Mini Nutritional Assessment; *SF* short-form; *LF* long-form; *MN* malnourished; *AR* at risk for malnutrition; *WN* well-nourished; *n.a* not applicable; *n.r.* not reported; *PFS* progression-free survival; *TTP* time to progression; *NL* The Netherlands; *UK* United Kingdom; *US* United States of America

Most of the studies [16, 33–37, 39, 41, 42, 44–47, 49–57, 59, 60, 62, 63, 65–67, 69, 73, 75–79, 82–87] were conducted in Northern, Western or Southern Europe, 5 studies [38, 40, 43, 48, 68] in North or South America and 8 [58, 61, 64, 70, 72, 74, 80, 81] in Eastern Asia.

The number of included patients ranged from 30 to 2972, mean/median age from 53 to 82 years. In 8 studies [40, 52–54, 56, 58, 69, 87] also patients < 65 years were included. In 3 of these studies [40, 53, 58] mean age was 65 years or lower.

The percentage of female patients in studies including both sexes (*N* = 52) ranged from 9.7–96.0%. Three studies only included patients with prostate cancer [57, 59, 73] and one study only patients with gynecologic cancer [70].

Almost half of the studies [16, 34–36, 40–43, 45, 48–50, 55, 60, 62, 63, 66–68, 75, 78, 80, 82, 84, 86] reported on patients with various types of cancer. Thirty studies [33, 37–39, 44, 46, 47, 51–54, 56–59, 61, 65, 69–74, 76, 77, 79, 81, 83, 85, 87] focused on a specific type, with lung [52–54, 56, 81, 87] and colorectal cancer [37, 39, 44, 46, 51, 65, 69, 76, 77] as the most common types. Fifteen studies [33, 39, 44, 52–58, 60, 76, 81, 84, 87] included only patients with advanced cancer, while 2 studies [57, 73] excluded patients with metastatic cancer. For studies reporting various cancer stages (*N* = 26), the percentage of patients with stage III and stage IV (metastatic) ranged from 15 to 56% and from 4 to 86%, respectively.

### MNA

In 30 studies [40, 42, 44, 46–49, 51–57, 60, 62, 64–66, 69–72, 78–80, 82–84, 87] the MNA-LF, in 20 studies [16, 38, 39, 43, 45, 50, 58, 59, 61, 63, 67, 68, 73–77, 81, 85, 86] the MNA-SF, and in 5 studies [33–37] a stepwise approach that considered both forms was used. One study [41] did not report the MNA-version.

All MNA-categories (malnourished, at risk of malnutrition and well-nourished) were reported in 25 studies [35, 38, 42, 43, 52–56, 58, 63–66, 68, 69, 73, 74, 78–81, 83, 86, 87] with prevalence of malnutrition ranging from 0 to 35.7% and of risk of malnutrition from 6.7–66.7%. Twenty-three studies [16, 33, 34, 36, 37, 39, 44–47, 49, 59–62, 67, 71, 75–77, 82, 84, 85] merged patients with malnutrition and at risk of malnutrition, and reported 27.0–85.0% being at least at risk, while 2 other studies [57, 72] merged patients at risk of malnutrition and well-nourished patients. Four studies reported a mean or median baseline MNA-score [40, 48, 51, 70], and 2 studies did not report concrete results [41, 50].

### Reported outcomes

Thirty-three studies investigated the association between MNA and mortality / (poor) overall survival, 3 reported progression-free survival, 2 time to progression, 11 treatment maintenance, 15 adverse treatment outcomes, 4 functional status or decline, 3 (health-related) quality of life (Table 2 and Additional file table 3a-f). Other outcomes were less often reported: length of hospital stay in 2 studies and falls, fatigue and unplanned admission in 1 study, each and are reported in the results section.

**Table 2:** Univariate and multivariable associations of MNA and the most frequent investigated health and treatment outcomes in patients with cancer

Study	N^**a**^	Type of cancer	Mean/ median follow-up^**b**^ (months)	Association with MNA and							
				Mortality/ poor survival	Progression-free survival/ Time to progression	Treatment maintenance/ -duration	Complications		Functional status/ -decline		Quality of Life
							Treatment toxicity	Postoperative complications	ADL	IADL	
Aaldriks 2011	202	Various	9	**+ +**		**+**					
Aaldriks 2013a	143	Colorectal	15	any CT: **+ +** adjuvant CT: **– –** palliative: **+ +**		**+ +**					
Aaldriks 2013b	55	Breast	16	**+ +**		**–**					
Aaldriks 2015	44	Various	46	**–**		**+ +**					
Aaldriks 2016	494	Various	17	**+ +**		**+ +**					
Allaire 2017	144	Bladder	0.2 or 2					**– –** **– –**			
Aparicio 2018	102	Colorectal	20.4	**–**	**–**						
Baier 2016	195	Various	6						**–**		
Boulahssass 2018	1050	Various	3.3	**+ +**							
Decoster 2016	193	Colorectal	2–3				Grade 4 HT: **– –** Grade 4 NHT: **– –**		**–**	**–**	
Decoster 2018	252	Colorectal	n.r.		**+ +**	**–**					
Decoster 2019	2972	Various	3								**+ +**
Dubruille 2015	90	Hematological	12	**–**							
Extermann 2012	n.a.^c^	Various	1				HT: **– –** NHT: **+ +**				
Frasca 2018	1264	Various	12 36 60	**+ +** **+ +** **– –**							
Ghosn 2017	100	Various	47.3	**–**							
Giannotti 2019	99	Colorectal	12	**–**							
Giannousi 2012	122	Lung	70	**+ +**							
Gioulbasanis 2011a	173	Lung	24	**+ +**	**+ +**						
Gioulbasanis 2011b	115	Lung	38.2	**+ +**							
Gioulbasanis 2012	114	Lung	24.3	**+ +**	**+ +**						
Gioulbasanis 2015	594	Various	27	**+ +**							
Goineau 2018	100	Prostate	2								**–**
Gu 2015	300	Renal cell	30.8	**+ +**			**– –**^e^				
Honecker 2018	160	Prostate	n.r.	**–**		**–**					
Hoppe 2013	299	Various	n.r.						**+**		
Kaibori 2016	71	Hepatocellular	n.r.					**–**			
Kenig 2015	75	Various	1					**–**			
Kenis 2017	439	Various	n.r.	**+ +**					**+ +**	**–**	
Kenis 2018	Cohort A: 763	Various	Cohort A: 61.4	**+ +**							
	Cohort B: 402		Cohort B: 45.7	**+ +**							
Kim 2014	98	Various	15.1			**+ +**					
Kristjansson 2010	182	Colorectal	20	**+ +**				**–**^f^			
Liuu 2020	1092	Various	15.3	**+**							
Lycke 2019	944	Various	12	**+ +**							
Martucci 2016	136	Various	12	**+ +**							
Mazzuca 2019	ProLYOtin^d^: 22	Colorectal	3				**+**				
	Placebo: 25										
Michaan 2020	120	Gynaecologic	> 4	**+ +**							
Molga 2020	98	Hematological	n.r.	**–**		**–**					
Naito 2016	93	Non-Hodgkin Lymphoma	n.r.	**–**							
Osborne 2017	178	Prostate	3				**–**				
Park 2015	70	Lymphomas	21.5	**+ +**		**+ +**					
Quinten 2019	Surgery: 741	Various	3								**+ +**
	Chemo: 683										**–**
Retornaz 2020	97	Colorectal	16.7	**–**			**–**				
Samuelsson 2019	49	Colorectal	≤0.1					**–**			
Scholtz 2018	517	Various	1					**+**			
Schütte 2015	51	Hepatocellular	7.3	**+**							
Shin 2012	64	Various	2.1				**–**				
Shiroyama 2017	30	Lung	n.r.				**+**				
Soubeyran 2012	348	Various	6	**+ +**							
Stauder 2020	147	Hematological	24	**+**							
van Deudekom 2019	102	Head & Neck	12	**+ +**							
van der Vlies 2019	99	Various	n.r.			**–**	**–**				
Vlachostergios 2013	103	Lung	38.2	**+ +**	**–**						
**Proportion of studies with significant results in multivariable analyses**				**22/27** **(=81%)**	**3/5** **(=40%)**	**5/8** **(=63%)**	**1/5** **(=20%)**	**0/2** **(=0%)**	**1/3** **(=33%)**	**0/2** **(=0%)**	**2/2** **(=100%)**

Legend: ^a^: Number of included patients; ^b^: median (range), mean ± SD or pre-defined follow-up time; ^c^: total: 518; ^d^: Highly purified, whey protein group; ^e^: at 30 days; ^f^: 1 month

+ +: MNA significantly associated with outcome in multivariable analyses

– –: MNA not significantly associated with outcome in multivariable analyses

+: MNA significantly associated with outcome in univariate regression analyses or by other statistical tests (e.g. chi-square), and no multivariable analyses reported or MNA not included in multivariable model

–: MNA not significantly (*p* < 0.05) associated with outcome in univariate analyses or by other statistical tests (e.g. chi-square)

*HT* hematologic toxicity; *NHT* non-hematologic toxicity; *(I)ADL* (instrumental) activities of daily living; *n.r*. not reported

### Mortality / (poor) overall survival

In 10 studies a specific follow-time point was reported (100 and 500 days, 6, 12, 24, 36 and 60 months), in 20 studies follow-up times varied with median follow-up times between 9 and 70 months. Mortality rates varied between 16% in 6 months and 94% in 38 months (29 studies). The mean/ median time for overall survival ranges from 5 to 38 months (9 studies) (Table 2, Additional file table 3a).

All studies analyzing the malnourished category separately (*N* = 7) report significant results with 3 to 8 times higher chance for mortality for malnourished compared to well-nourished patients [42, 53, 55, 56, 58, 68, 87]. In all of these studies, the chance for mortality was lower in patients at risk of malnutrition than in malnourished patients, but still significant in 4 studies [42, 53, 55, 56].

In 1 study reporting 12-, 36- and 60 months-mortality in patients with (risk of) malnutrition compared with well-nourished patients, significance was lost at 60 months [49]. In 12 of 18 studies with a combined malnutrition/ at risk of malnutrition group, the chance for mortality was also significantly increased [33, 35–37, 54, 63, 65, 82] compared to well-nourished patients in multivariable analyses. In a subgroup analysis in 1 of these studies, the relation remained only significant in patients receiving palliative chemotherapy but not in patients with adjuvant chemotherapy [37]. In 1 [74] of 2 studies [72, 74] the chance for mortality was significantly higher for patients with malnutrition when compared to those being at risk of malnutrition or well-nourished. One study [52] showed a significant association of MNA with mortality but did not report whether the continuous or categorical MNA-result was used for analysis, while another study showed also a significant association but used the MNA-score [67]. Six other studies only reported results from univariate analyses [51, 59, 72, 76, 79, 83].

### Progression-free survival and time to progression

Of 3 studies [39, 44, 87] examining progression-free survival in patients with either colorectal or lung cancer, only 1 [44] found the MNA to be predictive (Table 2, Additional file table 3b).

Two studies investigated the prognostic ability of MNA for time to progression of metastatic lung cancer [53, 56]. Both reported a higher chance for a longer time to progression for well-nourished patients when compared to patients at risk of malnutrition and malnourished patients in multivariable analyses.

### Treatment maintenance

Treatment maintenance was examined in 3 ways: not completing scheduled chemotherapy cycles, treatment discontinuation and treatment duration.

Not completing the scheduled cycles of chemotherapy was investigated in 7 studies [33–37, 74, 84] and those presenting adjusted analyses (*n* = 3) showed a significant higher chance for patients with (risk of) malnutrition compared to well-nourished patients [34, 36, 37] or malnourished patients compared to those who were well-nourished or at risk of malnutrition [74]. Two studies did not report an adjusted analysis [35, 84] and in 1 study a significant association could not be obtained in multivariable analysis [33] (Table 2, Additional file table 3c).

One [64] of 3 studies [59, 64, 71] focusing on treatment discontinuation reported a significantly higher chance for patients with malnutrition compared to those who were well-nourished or at risk of malnutrition.

One further study [44] focused on treatment duration and failed to show an association with MNA-result at baseline.

### Adverse treatment outcomes

Nine studies investigated the association between baseline MNA and treatment toxicity [46, 48, 58, 69, 73, 76, 80, 81, 84] (Table 2, Additional file table 3d). In only 1 of these studies [48], a significant higher risk for non-hematologic toxicity was shown for patients with (risk of) malnutrition compared to well-nourished patients, while for other toxicity outcomes (hematologic, acute radiotherapy or significant toxicity) MNA-result was not predictive [46, 73, 80] or not investigated in adjusted analyses [69, 76, 81, 84].

In all 6 studies reporting various kinds of postoperative complications, MNA did not maintain significant results or was not investigated in multivariable analyses [38, 61, 62, 65, 77, 78].

### Functional status/ decline

One study identified functional limitations defined as Barthel-ADL < 95 after 6 months in 10% of patients with various types of cancer and reported no significant association of this outcome with the baseline MNA-result in the unadjusted analysis (Chi^2^-test) [41] (Table 2, Additional file table 3e).

Functional decline in activities of daily living and instrumental activities of daily living was examined in 3 studies [46, 60, 63] with different tools and was not significantly associated with the MNA-result in all but 1 study, where the odds for ADL-decline was two-fold in patients with (risk of malnutrition) compared to well-nourished patients [63]. Another study in about 300 patients with various types of cancer did not conduct multivariable analyses [60].

### (health-related) quality of life

Three studies reported (Health-related) quality of life [57] (Table 2, Additional file table 3f). Until a follow-up of 2 months, quality of life declined in 30% of patients with localized advanced prostate cancer and a low prevalence of malnutrition at baseline (2%), but the study did not report adjusted analyses related to its association with baseline MNA [57]. In two studies [45, 75] reporting on patients with various types of cancer and a follow-up of 3 months, patients with (risk of) malnutrition had a significantly lower chance for a decline in health-related quality of life compared to well-nourished patients. In one of these studies, this effect was not maintained in in the multivariable analysis in patients receiving chemotherapy [75].

### Other outcomes

Two studies reported results on length of hospital stay investigated in univariate analyses [43, 77] . In 1 study [77], length of hospital stay was longer in patients with malnutrition while in the other study [43], nutritional status according to MNA did not show an association.

One study showed that MNA-score was predictive for fatigue evaluated by the Chalder Fatigue Scale (mean value at follow-up 26.8 ± 4.8; correlation coefficient *r* = − 0.52, *p* = 0.01) but not by the Brief Fatigue Inventory (mean value at follow-up 22.4 ± 23.7; correlation coefficient and *p*-value not reported) in chemotherapy-treated patients with various types of cancer and a mean age of 53 years [40].

In 1 study reporting a fall incidence of about 18% during 2–3 months, nutritional status was not a prognostic factor for patients with various kinds and stages of cancer (not significant in multivariable analysis) [86].

Another study including patients with various types of cancer reported a significant univariate association between MNA and unplanned (hospital) admissions but did not consider MNA for further multivariable analyses [66].

### Risk of Bias

The RoB of all studies was moderate to high (Additional file 2, table 3). Main sources of potential bias were residual confounding due to missing prespecified potential confounding variables (e.g. age, sex, performance status) in multivariable models.

## Discussion

In this systematic review, we investigated the prognostic significance of baseline nutritional status according to MNA regarding health and treatment outcomes in patients with cancer. In 56 studies included in our review, we found that, based on a moderate to high risk of bias, poor nutritional status is associated with a significantly higher risk for mortality / poor overall survival (22/27 studies), longer progression-free survival / time to progression (3/5 studies), worse treatment maintenance (5/8 studies) and (health-related) quality of life (2/2 studies) in multivariable analyses. Adverse treatment outcomes (1/7 studies) and functional decline (1/3 studies) were not significantly predicted by MNA in adjusted analyses while other outcomes were not investigated in multivariable analyses.

The **MNA** was originally developed to identify patients 65 years or older at risk of malnutrition irrespective of a specific disease [23, 25].

The **prevalence of malnutrition**, risk of malnutrition or their combination was 0–41%, 7–67%, and 28–67%, respectively – however not reported in all studies (Additional file 1, Table 1). We could not identify a trend for a higher or lower prevalence of malnutrition in studies including patients with a specific kind or stage of cancer as documented in a large cohort study from Italy including 1952 patients with various types and stages of cancer. There, a prevalence for malnutrition of 8.7% and risk of malnutrition of 42.4% was reported for all patients, but when stratified for cancer stage, both MNA-categories, malnutrition and risk of malnutrition were significantly higher in stage IV compared to stage I-III cancer [88]. A meta-analysis of studies including hospitalized patients older than 60 years with any disease, reported a prevalence for malnutrition of 22.0% (95%-CI: 18.9–25.2) and risk of malnutrition 45.6% (95%-CI: 42.7–48.6) [89]. Recently, a consensus for the diagnosis of malnutrition, the Global Leadership Initiative on Malnutrition (GLIM)-criteria, was published [90] and a few studies regarding nutritional status in patients with cancer are available. Prevalence rates for malnutrition according to GLIM were reported between 25.8 and 80% - depending on the criteria which were used for the diagnosis according to GLIM [91–93].

We could show that the **chance for mortality** was higher in patients being malnourished and at risk of malnutrition compared to well-nourished patients in the majority of studies (Table 2, Additional file table 3a). This is in line with 3 recently published systematic reviews also addressing the relation between malnutrition and mortality in patients with cancer [94–96]. While their approaches and search strategies differed with respect to inclusion of other screening tools and prespecified outcomes, there is an overlap of included MNA-studies. However, we could identify additional studies, so that our systematic review adds further evidence for the relationship between nutritional status assessed by MNA and mortality. Other systematic reviews with focus on a specific type of cancer (pancreatic, gastrointestinal) [97–99] or cancer stage (advanced) [100] reported that mortality risk / overall survival is predicted by nutritional status according to low body mass index, the Prognostic Nutritional Index, Controlling Nutritional status and phase angle [97–100]. For the Patient-Generated Subjective Global Assessment, which is also often used and recommended for nutritional screening in patients with cancer, several primary studies investigated the association with mortality/ overall survival and showed conflicting results with a majority of studies predicting a higher risk [101–104]. Two studies investigated the association between malnutrition according to the GLIM-criteria and mortality/ poor survival and both could show significant results [91, 92]. Future studies should investigate the application and prognostic abilities of these criteria. Additionally, an analysis including several cohorts of patients with cancer could show that the risk for mortality was higher in patients with lower body mass index and higher weight loss [105]. In one study (which was excluded), machine learning algorithms were used to predict early death in older patients with cancer [106]. Questionnaire items from the comprehensive geriatric assessment were selected by artificial intelligence and the MNA-SF remained in the predictive model. Such studies might be used in future to gain further knowledge of the prognostic factors in patients with cancer. Regarding other diseases, a meta-analysis found nutritional status according to MNA being predictive for mort ality in patients with heart failure [107].

Besides mortality risk, **time to progression and progression-free survival** are often used endpoints in clinical trials to evaluate the efficacy of anti-cancer treatment, since the treatment intention is either curation or a longer survival with a higher quality of life [108], but were only rarely investigated in relation to MNA. We found evidence that a poor MNA-result is predictive for a shorter time to progression / progression-free survival (Table 2, Additional file table 3b). These endpoints are mostly not clearly defined, but it is generally agreed among experts that time to progression reflects the time to cancer progression whereas progression-free survival also includes death from any cause [109, 110]. However, it is discussed whether these endpoints are meaningful outcomes in cancer research, since a recent systematic review, including about 14,000 adult patients until 93 years with various kinds of cancer, showed that a prolonged progression-free survival is not associated with a higher health-related quality of life [111]. The association between a poorer nutritional status and a higher risk for a shorter progression-free survival was also shown in a recent meta-analysis investigating the prognostic ability of the Prognostic Nutritional Index in patients with hepatocellular carcinoma [112]. For other tools, primary studies found a shorter progression-free survival significantly predicted by nutritional status assessed by the Geriatric Nutritional Risk Index or the Controlling Nutritional Status Score in different types of cancer [113, 114], but systematic reviews are lacking.

When patients with cancer had poor MNA at baseline, **treatment maintenance** was poorer but treatment duration (1 study) was not shorter (Table 2, Additional file table 3c). Main reasons for poorer maintenance were toxicity, cancer progression and insufficient therapeutic effect [33–36].

In included studies investigating **toxicity** as a separate outcome, a significant association with MNA-result at baseline was not found. Only non-hematologic toxicity was predicted by a poorer nutritional status according to MNA in 1 study [48] (Table 2, Additional file table 3d). Also, **complications after surgery** were not predicted by MNA (Table 2, Additional file table 3d). This is in line with results of other systematic reviews including patients with various kinds of cancer that showed a lower chance of treatment-related adverse events by geriatric assessment components only according to functional status, cognition and depression but not by nutritional status according to various definitions [95, 115, 116]. In contrast, in adult patients undergoing joint arthroplasty or hip fracture surgery, malnutrition defined by serologic markers (e.g. albumin, lymphocyte count, transferrin) was predictive for a higher risk of postoperative outcomes, such as wound complications [117, 118]. One study in older hip-fracture patients showed that patients with (risk of) malnutrition patients – according to MNA-SF – were at higher risk for postoperative delirium compared to well-nourished patients [119]. In another study, a significant association between malnutrition and chemotherapy related toxicity could be showed for the Patient-Generated Subjective Global Assessment but not for the Nutritional Risk Index [103]. To clarify these conflicting results, further studies are required in patients with cancer.

Only 1 of 3 studies predicted functional decline in basic activities of daily living by poor MNA-result and all 3 studies failed to predict a decline in instrumental activities of daily living (Additional file, table 3e). In older hospitalized patients with various diseases, nutritional status according to Short Nutritional Assessment Questionnaire was also not related to functional decline [120] but a moderate association was found for MNA in older people from different settings (i.e. community-dwelling, acute, subacute or residential care) [121]. A systematic review of studies including older hospitalized patients with various diseases revealed that baseline functional and cognitive status as well as social support were more important to predict functional outcomes than nutritional status [122]. These results demonstrate the need for further studies regarding the association between MNA and functional decline in patients with cancer.

For **(health-related) quality of life**, 1 study that was identified by our systematic literature search was only small, including patients with prostate cancer. Only 2% were malnourished and unfortunately no adjusted analysis was reported [57]. Two other studies that we also included could show a lower chance for a decline in health-related quality of life for patients with (risk of) malnutrition [45, 75] (Table 2, Additional file table 3f). This finding might be explained by the already poor quality of life at baseline or, in other words that after anticancer therapy the chance for an improvement in quality of life was higher for patients with (risk of) malnutrition compared to well-nourished patients with already better quality of life.

Regarding length of hospital stay (2 studies), fatigue, falls and unplanned admissions (1 study each), only a very small number of studies investigated the association with baseline MNA with no multivariable analyses, and more studies are needed also in this regard to draw any conclusion.

Several limitations of the included studies need to be considered. First, risk of bias was judged as moderate to high in all included studies, which is in contrast to other systematic reviews reporting a low to moderate risk of bias [94, 95]. Our rating is mainly explained by insufficient consideration of potential confounders – which have been predefined by 2 reviewers (cancer stage, type of cancer, sex, age, performance status, co-morbidity) – in multivariable analyses of primary studies to minimize the risk of residual confounding which is generally one of the most relevant limitations of observational studies [123–125]. Second, in several studies [33, 34, 37, 42, 53, 56, 58, 61, 64, 68, 74, 80, 82, 87], effect estimates had relatively wide confidence intervals and this imprecision should be considered when interpreting these results. Reasons for imprecisions might be an insufficient number of participants or malnourished patients. Third, follow-up times differed widely between the studies and only a few defined or reported a specific time point for outcome assessment. Mostly only vague information about follow-up times, such as a mean overall survival, was provided. Thus, conclusions for a specific time-frame cannot be drawn.

Furthermore, we included articles that report on study populations recruited from the same hospitals within a recruitment time from 2004 to 2010 [33–37]. All patients were treated by chemotherapy. We did not exclude one of these studies, since 2 publications focused on a specific type [33, 37] and the other 3 publications included various types of cancer [34–36] with 1 study focusing on patients with different types of non-Hodgkin Lymphoma [34] and 1 study with a shorter recruitment period [35]. Although reported results differed, this overlap should be kept in mind.

### Strengths

Main strength of this systematic review is its strict methodology which followed the PRISMA guideline [31]. We conducted an extensive literature search without any language restrictions and did not specify search terms for outcomes to integrate all health and treatment outcomes that were investigated in primary studies. Each review step (screening, data extraction and RoB assessment) was piloted and performed by 2 reviewers independently. Additionally, we focused on 1 screening tool to minimize heterogeneity due to assessment. As part of the assessment of RoB, our rating of the confounding domain was strict and other reviewers might rate differently – but this is a general problem with RoB rating.

### Limitations

The databases we used have their major focus on journals from the US and Europe and journals from other regions might not have been identified by our exhaustive systematic literature search. Therefore, language bias cannot be excluded although we did not restrict our search to specific languages.

The large heterogeneity of included studies regarding samples, treatments and outcome assessments should also be considered when interpreting our results. However, despite this heterogeneity, a rather stable relation between MNA result and several outcomes was observed.

### Implications for research

Large, prospective and registered cohort studies should be conducted to strengthen our results, which are based on heterogeneous samples and outcomes. In addition, future studies that investigate the comparison of the prognostic ability of different nutritional screening/ assessment tools (such as the MNA, the Patient-Generated Subjective Global Assessment or the Nutritional Risk Screening 2002) or criteria (such as the GLIM-criteria) are needed. Publications should follow the respective guidelines provided by the Enhancing the QUAlity and Transparency Of health Research (EQUATOR) network (https://www.equator-network.org/reporting-guidelines/) to further standardize reporting of studies.

### Implications for practice

Based on our observation of negative health and treatment outcomes in patients with poor MNA-result and in light of available effective nutritional interventions, health care professionals should be aware of nutritional status and should support and engage patients to improve their nutritional status before and during anti-cancer therapy.

## Conclusions

According to available studies, MNA-result predicts risk of mortality/survival, progression-free survival/time to progression, treatment maintenance and (health-related) quality of life in patients with cancer and does not predict adverse treatment outcomes and functional status/ decline. For other outcomes the results are less clear. A high risk of bias should however be considered. To verify these findings, further studies with good control of potential biases are needed.

## Supplementary information

**Additional file 1: Table S1.** Search strategy Medline (via Ovid). Table S3a: Results on mortality and poor overall survival (OS) (*N* = 33). Table S3b: Results on disease progression (progression-free survival (PFS) and time to progression (TTP)) (*N* = 5). Table S3c: Results on treatment maintenance or duration (*N* = 11). Table S3d: Results on adverse treatment outcomes (*N* = 15). Table 3e: Results functional status/ - decline (*N* = 4). Table S3f: Results (health-related) quality of life (*n* = 3).

## Data Availability

Data sharing is not applicable to this article as no datasets were generated or analysed during the current study.

## Abbreviations

HR: Hazard ratio
LF: Long-form
MNA: Mini-Nutritional Assessment
OR: Odds ratio
PRISMA: Preferred Reporting Items for Systematic Reviews and Meta-Analyses
RoB: Risk of bias
SF: Short-form
