# Supplementary material for: Nutritional status according to the mini nutritional assessment (MNA)® as potential prognostic factor for health and treatment outcomes in patients with cancer – a systematic review
Source: BMC Cancer. 2020 Jun 26;20:594. doi: 10.1186/s12885-020-07052-4 (PMC7318491; doi:10.1186/s12885-020-07052-4)
Supplement: Supplementary file 1 — Additional file 1: Table S1. Search strategy Medline (via Ovid). Table S3a: Results on mortality and poor overall survival (OS) (N = 33). Table S3b: Results on disease progression (progression-free survival (PFS) and time to progression (TTP)) (N = 5). Table S3c: Results on treatment maintenance or duration (N = 11). Table S3d: Results on adverse treatment outcomes (N = 15). Table 3e: Results functional status/ - decline (N = 4). Table S3f: Results (health-related) quality of life (n = 3). [file 12885_2020_7052_MOESM1_ESM.docx]

Additional file, table 1: Search strategy Medline (via Ovid)

| **#** | **Searches** |
| --- | --- |
| 1 | exp Neoplasms/ |
| 2 | neoplas*.ti,ab,kf. |
| 3 | cancer*.ti,ab,kf. |
| 4 | carcinom*.ti,ab,kf. |
| 5 | tumo?r.ti,ab,kf. |
| 6 | oncolog*.ti,ab,kf. |
| 7 | blastoma*.ti,ab,kf. |
| 8 | sarcoma*.ti,ab,kf. |
| 9 | lymphoma*.ti,ab,kf. |
| 10 | leuk?emia*.ti,ab,kf. |
| 11 | metasta*i*.ti,ab,kf. |
| 12 | or/1-11 |
| 13 | nutrition assessment/ |
| 14 | Geriatric Assessment/ |
| 15 | mini* nutrition* assessment.ti,ab,kf. |
| 16 | mna*.ti,ab,kf. |
| 17 | g8*.ti,ab,kf. |
| 18 | geriatric* 8.ti,ab,kf. |
| 19 | g-8*.ti,ab,kf. |
| 20 | comprehensive geriatric assessment.ti,ab,kf. |
| 21 | cga.ti,ab,kf. |
| 22 | (nutrition* adj3 screen*).ti,ab,kf. |
| 23 | or/13-22 |
| 24 | Malnutrition/ |
| 25 | Protein-Energy Malnutrition/ |
| 26 | malnutrition.ti,ab,kf. |
| 27 | malno?ris*.ti,ab,kf. |
| 28 | undernutrition.ti,ab,kf. |
| 29 | underno?ris*.ti,ab,kf. |
| 30 | nutritional status.ti,ab,kf. |
| 31 | mini* nutrition* assessment.ti,ab,kf. |
| 32 | mna*.ti,ab,kf. |
| 33 | g8*.ti,ab,kf. |
| 34 | geriatric* 8.ti,ab,kf. |
| 35 | g-8*.ti,ab,kf. |
| 36 | function*.ti,ab,kf. |
| 37 | or/24-36 |
| 38 | 12 and 23 and 37 |
| 39 | limit 38 to yr="1994 -Current" |

Legend: Ovid MEDLINE(R) Epub Ahead of Print, In-Process & Other Non-Indexed Citations, Ovid MEDLINE(R) Daily, Ovid MEDLINE and Versions(R);

Search Date: June 02, 2017 and September 21, 2018

Search fields: ti: Title; ab: Abstract; kf: keyword heading word;

Exp (…)/ MeSH-Term including expansion to subcategories

(…)/ MeSH-Term

adjX Defined Adjacency

? Optional Wildcard

* Unlimited Truncation

Additional file, table 2: Risk of Bias of Included Studies

| **Author/ Year** | **Risk of Bias (low/ moderate/ high)** | | | | | |
| --- | --- | --- | --- | --- | --- | --- |
|  | **Study Participation** | **Study Attrition** | **Prognostic Factor Measurement** | **Outcome Measurement** | **Study Confounding** | **Statistical Analysis and Reporting** |
| Aaldriks 2011 |  |  |  |  |  |  |
| Aaldriks 2013a |  |  |  |  |  |  |
| Aaldriks 2013b |  |  |  |  |  |  |
| Aaldriks 2015 |  |  |  |  |  |  |
| Aaldriks 2016 |  |  |  |  |  |  |
| Allaire 2017 |  |  |  |  |  |  |
| Aparicio 2018 |  |  |  |  |  |  |
| Araujo 2017 |  |  |  |  |  |  |
| Baier 2016 |  |  |  |  |  |  |
| Boulahssass 2018 |  |  |  |  |  |  |
| D’Almeida 2020 |  |  |  |  |  |  |
| Decoster 2016 |  |  |  |  |  |  |
| Decoster 2018 |  |  |  |  |  |  |
| Decoster 2019 |  |  |  |  |  |  |
| Dubruille 2015 |  |  |  |  |  |  |
| Extermann 2012 |  |  |  |  |  |  |
| Frasca 2018 |  |  |  |  |  |  |
| Ghosn 2017 |  |  |  |  |  |  |
| Giannotti 2019 |  |  |  |  |  |  |
| Giannousi 2012 |  |  |  |  |  |  |
| Gioulbasanis 2011a |  |  |  |  |  |  |
| Gioulbasanis 2011b |  |  |  |  |  |  |
| Gioulbasanis 2012 |  |  |  |  |  |  |
| Gioulbasanis 2015 |  |  |  |  |  |  |
| Goineau 2018 |  |  |  |  |  |  |
| Gu 2015 |  |  |  |  |  |  |
| Honecker 2018 |  |  |  |  |  |  |
| Hoppe 2013 |  |  |  |  |  |  |
| Kaibori 2016 |  |  |  |  |  |  |
| Kenig 2015 |  |  |  |  |  |  |
| Kenis 2017 |  |  |  |  |  |  |
| Kenis 2018 |  |  |  |  |  |  |
| Kim 2014 |  |  |  |  |  |  |
| Kristjansson 2010 |  |  |  |  |  |  |
| Liuu 2020 |  |  |  |  |  |  |
| Lycke 2019 |  |  |  |  |  |  |
| Martucci 2016 |  |  |  |  |  |  |
| Mazzuca 2019 |  |  |  |  |  |  |
| Michaan 2020 |  |  |  |  |  |  |
| Molga 2020 |  |  |  |  |  |  |
| Naito 2016 |  |  |  |  |  |  |
| Osborne 2017 |  |  |  |  |  |  |
| Park 2015 |  |  |  |  |  |  |
| Quinten 2019 |  |  |  |  |  |  |
| Retornaz 2020 |  |  |  |  |  |  |
| Samuelsson 2020 |  |  |  |  |  |  |
| Scholtz 2018 |  |  |  |  |  |  |
| Schütte 2015 |  |  |  |  |  |  |
| Shin 2012 |  |  |  |  |  |  |
| Shiroyama 2017 |  |  |  |  |  |  |
| Soubeyran 2012 |  |  |  |  |  |  |
| Stauder 2020 |  |  |  |  |  |  |
| van Deudekom 2020 |  |  |  |  |  |  |
| van der Vlies 2019 |  |  |  |  |  |  |
| Vande Walle 2014 |  |  |  |  |  |  |
| Vlachostergios 2013 |  |  |  |  |  |  |

Additional file, table 3a: Results on mortality and poor overall survival (OS) (N=33)

| **Author/ Year** | **Follow-up (months)^a^** | **Died (%) or**  **median OS [95%-CI] (months)** | **Chance for mortality/ poor OS (HR/ OR [95%-CI])** | | |
| --- | --- | --- | --- | --- | --- |
|  |  |  | **Comparison**  **MN vs WN** | **Comparison**  **AR vs WN** | **Comparison**  **MN/AR vs WN** |
| Aaldriks 2011 | 9 (1-33) | ~65% |  |  | HR **2.54 [1.55-4.15]** |
| Aaldriks 2013a | 15 (0.5-62) | 53% |  |  | for any CT  HR **2.54 [1.49-4.33]** |
|  |  |  |  |  | for adjuvant CT: 1.04 [0.20-5.25] |
|  |  |  |  |  | for palliative CT: **2.76 [1.60-4.77]** |
| Aaldriks 2013b | 16 ± 13.7 | 75% |  |  | HR **3.05 [1.44-6.45]** |
| Aaldriks 2015 | 46 (0-101) | 65% |  |  | HR 1.46 [0.56-3.78] |
| Aaldriks 2016 | 17 (1-101) for all  61 (44-101) for survivors | 80% |  |  | HR **1.86 [1.48-2.34]** |
| Aparicio 2018 | 20.4 (11.8-31.2)^b^ | 20.4 [16.8-23.9] |  |  | HR 1.32 [0.72-2.43] |
| Boulahssass 2018 | 100 days | 20% | OR **8.1 [2.1-31.1]** | OR **4.4 [1.2-16.6]** |  |
| Dubruille 2015 | 12 | 28% |  |  | n.s.^c^ |
| Frasca 2018 | 12 | 35.3%^d^ |  |  | 12 months: HR **2.97 [2.33-3.78]** |
|  | 36 |  |  |  | 36 months: HR **2.24 [1.82-2.77]** |
|  | 60 |  |  |  | 60 months: HR 0.73 [0.43-1.24] |
| Ghosn 2017 | 47.3 | 55.0% |  |  | HR 0.65, *p*=0.18^e^ |
| Giannotti 2019 | 12 | 19% |  |  | n.s.^f^ |
| Giannousi 2012 | 70 | 11.1 [9.7-12.5] |  |  | HR **1.65, *p*=0.03**^g^ |
| Gioulbasanis 2011a | 24 | n.r. | HR **3.7 [1.89-7.27]** | HR **1.24 [1.34-3.75]** |  |
| Gioulbasanis 2011b | 38.2 (0.1-49.4) | n.r. |  |  | HR **1.64 [1.01-2.64]** |
| Gioulbasanis 2012 | 24.3 (0.1-33.5) | 4.6 (0.1-17.8)^h^ | HR **4.69 [2.19-10.08]** | HR **3.27 [1.68-6.37]** |  |
| Gioulbasanis 2015 | 27.0 (23.3-30.5) | 11.1 [9.7-12.5] | HR **2.58 [1.9-3.51]** | HR **1.78 [1.44-2.21]** |  |
| Gu 2015 | 30.8 | 62% | HR **2.78 [1.51-6.1]** | HR 1.32 [0.89-1.97] |  |
| Kenis 2017 | n.r. (median survival time 21.1) | 72% |  |  | HR **1.54** **[1.12-2.08]**^i^ |
| Kenis 2018 | Cohort A: 61.4 (0.7-75.6) | Cohort A: 62%; 33.2 (0.09-75.6)^h^ |  |  | Cohort A: **1.85 [1.51-2.27]** ^g,k^ |
|  | Cohort B: 45.7 (7.3-54.5) | Cohort B: 53%; 37.6 (0.16-54.5)^h^ |  |  | Cohort B: **1.64 [1.20-2.17]** ^g,k^ |
| Kristjansson 2010 | 20 | 26% |  |  | HR **2.39 [1.24-4.61]** |
| Liuu 2020 | 15.3 (6.4–29.1) | 60% (of those, 67% related to cancer) |  |  | no adjusted analysis |
| Lycke 2019 | 12 | 36% |  |  | **β=−0.147; p=0.000**^l^ |
| Martucci 2016 | 12 | 31.6% | OR **5.59 [1.8-17.3]** | OR 2.61 [0.8-8.2] |  |
| Michaan 2020 | >4 | 48% |  |  | **[1.10–4.41]**^m^ |
| Molga 2020 | n.r. | 59%; 22.9 |  |  | 1.68 [0.73-3.87]^g^ |
| Naito 2016 | n.r. | n.r. |  |  | n.s.^f,j^ |
| Park 2015 | 21.5 | 28.6% |  |  | OR **4.4 [1.7-11.5]**^i^ |
| Retornaz 2020 | 500 days | 30% |  |  | n.s.^f^ |
| Schütte 2015 | 218 ± 136 days | 23.5% |  |  | n.s.^c^ |
| Soubeyran 2012 | 6 | 16.1% |  |  | OR **2.77 [1.24-6.18]** |
| Stauder 2020 | 24 | 61% |  |  | no adjusted analysis |
| van Deudekom 2019 | 12 | 42%; 6.3 |  |  | **2.55 [1.23-5.26]** |
| Vlachostergios 2013 | 38.2 (0.1-49.4) | 94%; 9.2 ± 1.1^a^ | HR **2.84 [1.27-6.35]** | HR 1.55 [0.8-3.0] |  |

Legend: ^a^median (range), mean ± SD or pre-defined follow-up time; ^b^interquartile range; ^c^not significant in multivariable models, HR/OR not reported; ^d^died during study; ^e^comparison not known; ^f^not included in multivariable model; ^g^not reported if MNA-group or -score was used; ^h^range; ^i^inverse value of HR 0.65 [0.48-0.89] for longer OS**;** ^j^MN vs WN/AR; ^k^inverse value for HR 0.54 [0.44-0.66] and 0.61 [0.46-0.83] for longer OS; ^l^MNA-score; ^m^no OR/HR reported, only CI

AR: At risk for malnutrition; MN: malnourished; WN: well-nourished; HR: hazard ratio; OR: odds ratio; CI: confidence interval; CT: chemotherapy; n.r.: not reported; n.s.: not significant; *p*: *p*-value

Additional file, table 3b: Results on disease progression (progression-free survival (PFS) and time to progression (TTP)) (N=5)

| **Author/ Year** | **Follow-up (months)^a^** | **median PFS or TTP [95%-CI] (months)** | **Chance for longer PFS or TTP (HR [95%-CI])** | | |
| --- | --- | --- | --- | --- | --- |
|  |  |  | **Comparison**  **WN vs MN** | **Comparison**  **WN vs AR** | **Comparison**  **WN vs MN/AR** |
| *Progression-free survival* | | | | | |
| Aparicio 2018 | 20.4 (11.8-31.2)^b^ | 9.2 [7.7-10.6] |  |  | 0.90 [0.52-1.56] |
| Decoster 2018 | n.r. | 8.9 [7.9-9.9] |  |  | ***p*=0.0014** |
| Vlachostergios 2013 | 38.2 (0.1-49.4) | 4.2 ± 3.9^c^ | n.s.^d^ | n.s.^d^ |  |
| *Time to progression* | | | | | |
| Gioulbasanis 2011a | 24.0 (0.1-33.5) | n.r. | **3.32 [1.87-5.87]** | **1.67 [1.06-2.64]** |  |
| Gioulbasanis 2012 | 24.3 (0.1-33.5) | 4.2 (0.3-12.7)^e^ | **2.35 [1.48-7.92]** | **3.43 [1.16-4.76]** |  |

Legend: ^a^median (range); ^b^interquartile range; ^c^mean ± standard deviation; ^d^not significant in multivariable model, HR not reported; ^e^range

AR: At risk for malnutrition; MN: malnourished; HR: hazard ratio; CI: confidence interval; n.r.: not reported; *p*: *p*-value

Additional file, table 3c: Results on treatment maintenance or duration (N=11)

| **Author/ Year** | **Follow-up (months)^a^** | **% of patients failing to complete therapy or with treatment changes/**  **treatment duration** | **Chance for not completing therapy (OR [95%-CI])**  **Comparison**  **MN/AR vs WN** |
| --- | --- | --- | --- |
| Aaldriks 2011 | 9 (1-33) | 38.5 | no adjusted analysis |
| Aaldriks 2013a | 15 (0.5-62) | 28.3 | **3.45 [1.23-9.09]**^b^ |
| Aaldriks 2013b | 16 ± 13.7 | 29.1 | 1.03 [0.26-4.16] |
| Aaldriks 2015 | 46 (0-101) | 27.3 | **8.29 [1.24-55.6]** |
| Aaldriks 2016 | 17 (1-101) | 29.5 | **2.30 [1.48-2.34]** |
| Decoster 2018 | n.r. | treatment duration: n.r. | treatment duration: n.s.^d^ |
| Honecker 2018 | n.r. | 56.8 | n.s.^d^ |
| Kim 2014 | 15.1 | 30.6 | **5.03 [1.50-16.87]**^e^ |
| Molga 2019 | n.r. | n.s. | no adjusted analysis |
| Park 2015 | 21.5 | 33.8 | **6.2 [1.8-21.3]**^e^ |
| van der Vlies 2019 | n.r. | 47.5 | n.s.^f^ |

Legend: ^a^median (range) or mean ± SD; ^b^inverse values of OR 0.29 [0.11-0.81] for completing ≥4 (vs <4) CTC; ^c^not entered in multivariable model, OR not reported; ^d^OR not reported; ^e^MN vs WN/AR; ^f^not associated in univariate analyses

MN: malnourished; AR: at risk for malnutrition; WN: well-nourished; CTC: cycles of chemotherapy; CT: chemotherapy; OR: odds ratio; CI: confidence interval; n.r.: not reported; n.s.: not significant

Additional file, table 3d: Results on adverse treatment outcomes (N=15)

| **Author/ Year** | **Follow-up (months)** | **% of patients with adverse treatment outcome** | **Chance for adverse treatment outcome (OR [95%-CI])** | | |
| --- | --- | --- | --- | --- | --- |
|  |  |  | **Comparison**  **MN vs WN** | **Comparison**  **AR vs WN** | **Comparison**  **MN/AR vs WN** |
|  |  |  |  | | |
| *Treatment toxicity^a^* | | | | | |
| Decoster 2016 | 2-3 | Grade 3/4 HT: 5.5  Grade 3/4 NHT: 19.3 |  |  | HT: n.s.^b^  NHT**:** 3.03 [0.79-11.11]^d^ |
| Extermann 2012 | 1 | Grade 4 HT: 32.0  Grade 3/4 NHT: 56.0 |  |  | HT: 1.01 [0.94-1.09]^c,d^  NHT: **1.37 [1.11-1.67]**^c,d^ |
| Gu 2015 | 30 days | Grade 3/4: 32.7 | 0.64 [0.32–1.28] | 1.62 [0.49–5.70] |  |
| Mazzuca 2019 | 3 | Grade ≥2 CTCAE v4.0 Hematological: 23.0%  Grade ≥2 CTCAE v4.0 Gastrointestinal: 47.0% |  |  | no adjusted analysis |
| Osborne 2017 | 3 | Acute RTT: 32.7 |  |  | 1.14; *p*=0.50^d^ |
| Retornaz 2020 | 500 days | Grade 3/4 HT: 34.0  Grade 3/4 NHT: 44.3 |  |  | n.s. |
| Shin 2012 | 9 weeks | ST: 25.0 | 4.35 [0.22-84.64] | 2.72 [0.24-30.73] |  |
| Shiroyama 2017 | n.r. | Grade 3/4 HT: n.r.  Grade 3/4 NHT: n.r. |  |  | no adjusted analysis |
| van der Vlies 2019 | n.r. | Grade 3: 53.5 |  |  | n.s.^e^ |
| *Postoperative complications* | | | | | |
| Allaire 2017 | 7 days  90 days | -  79.0^f^ | 1.22 [0.68-2.17]  1.23 [0.67-2.23] | 1.08 [0.77-1.54]  1.14 [0.80-1.62] |  |
| Kaibori 2016 | n.r. | 25.4 |  |  | 2.08 [0.33-13.33]^g^ |
| Kenig 2015 | 30 days | All: 50.7  Major: 24.0 |  |  | no adjusted analysis |
| Kristjansson 2010 | 1 | n.r. |  |  | n.s.^g^ |
| Samuelsson 2019 | 1-3 days | 22.4 |  |  | no adjusted analysis |
| Scholtz 2018 | 1 | 25.5^h^ |  |  | n.s.^b^ |

Legend: ^a^as defined by study authors; ^b^not significant in multivariable analysis, OR not reported; ^c^inverse values for negative outcome; ^d^comparison unknown; ^e^not included in multivariable analyses; ^f^any complication (gastrointestinal complications were most frequent); study reports also results on gastrointestinal, infectious, wound, cardiac, low and high grade complications. All results from multivariable analyses not significant except for infectious complications after 90 days for AR vs WN (0.60 [0.37-0.98]); ^g^MN vs WN/AR; ^h^major and higher grade

AR: at risk for malnutrition; MN: malnourished; OR: Odds ratio; CI: confidence interval; r: correlation coefficient; n.r.: not reported; n.s.: not significant; *p*: *p*-value; HT: hematologic toxicity; NHT: non-hematologic toxicity; RTT: radiotherapy toxicity; ST: significant toxicity; BFI: Brief Fatigue Inventory, range 0-90; CFS: Chalder Fatigue Scale, range 0-33

Additional file, table 3e: Results functional status/ - decline (N=4)

| **Author/ Year** | **Follow-up (months)** | **% of patients with** | | **Chance for** | | |
| --- | --- | --- | --- | --- | --- | --- |
|  |  | **functional decline in ADL^a^** | **functional decline in IADL^a^** | **ADL < 95** | **functional decline (OR [95%-CI])**  **in ADL**  **Comparison**  **MN/AR vs WN** | **functional decline (OR [95%-CI]) in IADL**  **Comparison**  **MN/AR vs WN** |
|  |  | **ADL<95** | |  |  |  |
| Baier 2016 | 6 | 9.7 | | n.s.^b^ |  |  |
| Decoster 2016 | 2-3 | 18.0 | 37.0 |  | n.s.^b^ | n.s.^c^ |
| Hoppe 2013 | n.r. | 16.7 | - |  | n.s.^c^ | - |
| Kenis 2017 | 2-3 | 19.9 | 41.3 |  | **2.02 [1.10-3.71]** | n.s.^c^ |

Legend: ^a^as defined by study authors; ^b^ADL<95; not significant in multivariable analysis, OR not reported; ^c^not included in multivariable analysis

(I)ADL: (instrumental) activities of daily living; MN: malnourished; AR: at risk for malnutrition; MN: malnourished; OR: odds ratio; CI: confidence interval; n.r.: not reported

Additional file, table 3f: Results (health-related) quality of life (n=3)

| **Author/ Year** | **Follow-up (months)** | **% of patients with**  **Decline in QoL** | **Chance for**  **Decline (OR [95%-CI]) in QoL**  **Comparison**  **MN/AR vs WN** |
| --- | --- | --- | --- |
| Decoster 2019 | 3 | 28.2 | **0.78 [0.54-0.99]** |
| Goineau 2018 | 2 | 30 | n.s.^a^ |
| Quinten 2019 | 3 | Surgery: 23.5; | Surgery: **0.67 [0.46-0.98]** |
|  |  | Chemotherapy: 36.9 | Chemotherapy: 1.08 [0.72-1.62] |

Legend: ^a^not included in multivariable analysis; p>0.5 in univariate analysis

QoL: Quality of Life; MN: malnourished; AR: at risk for malnutrition; MN: malnourished; OR: odds ratio; CI: confidence interval; n.s.: not significant
